# VOC breath profile in spontaneously breathing awake swine during Influenza A infection

**DOI:** 10.1038/s41598-018-33061-2

**Published:** 2018-10-05

**Authors:** Selina Traxler, Ann-Christin Bischoff, Radost Saß, Phillip Trefz, Peter Gierschner, Beate Brock, Theresa Schwaiger, Claudia Karte, Ulrike Blohm, Charlotte Schröder, Wolfram Miekisch, Jochen K. Schubert

**Affiliations:** 1 Department of Anaesthesiology and Intensive Care, Rostock University Medical Center, ROMBAT, Schillingallee 35, 18057 Rostock, Germany; 2 Department of Experimental Animal Facilities and Biorisk Management, Friedrich-Loeffler-Institute, Südufer 10, 17493 Greifswald- Insel Riems, Germany; 3 Institute of Diagnostic Virology, Friedrich-Loeffler-Institute, Südufer 10, 17493 Greifswald-Insel Riems, Germany; 4 Institute of Immunology, Friedrich-Loeffler-Institute, Südufer 10, 17493 Greifswald-Insel Riems, Germany

## Abstract

Influenza is one of the most common causes of virus diseases worldwide. Virus detection requires determination of Influenza RNA in the upper respiratory tract. Efficient screening is not possible in this way. Analysis of volatile organic compounds (VOCs) in breath holds promise for non-invasive and fast monitoring of disease progression. Breath VOC profiles of 14 (3 controls and 11 infected animals) swine were repeatedly analyzed during a complete infection cycle of Influenza A under high safety conditions. Breath VOCs were pre-concentrated by means of needle trap micro-extraction and analysed by gas chromatography mass spectrometry before infection, during virus presence in the nasal cavity, and after recovery. Six VOCs could be related to disease progression: acetaldehyde, propanal, n-propyl acetate, methyl methacrylate, styrene and 1,1-dipropoxypropane. As early as on day four after inoculation, when animals were tested positive for Influenza A, differentiation between control and infected animals was possible. VOC based information on virus infection could enable early detection of Influenza A. As VOC analysis is completely non-invasive it has potential for large scale screening purposes. In a perspective, breath analysis may offer a novel tool for Influenza monitoring in human medicine, animal health control or border protection.

## Introduction

Influenza is one of the most common causes of virus diseases worldwide. In the US, 74.5% of hospitalizations were due to Influenza A and more than 47,000 human samples were tested positive during winter 2015/2016^1^. Almost 1000 people died due to Influenza during winter 2017/18 in Germany (https://influenza.rki.de, 15.04.2018).

Influenza is caused by an RNA virus from the *Orthomyxoviridae* family which is differentiated into three types: A, B, and C. Influenza A species are classified referred to the envelope glycoproteins hemagglutinin and neuraminidase. Depending on the combination of these proteins, Influenza A is divided in many subtypes, e.g. H1N1 or H3N2^2–4^. During simultaneous infection of one cell by two of these subtypes a gene shift between the two different virus genomes can occur. In this way, new virus subtypes can arise with novel genetic and biological features. Since natural reservoirs for Influenza A are humans, water birds, and swine, such re-assortments can also occur between zoonotic and human subtypes^2,4,5^. These facts still hamper diagnosis, development of vaccine development, and therapy of Influenza A infections^4^.

During recent years numerous studies have been undertaken using breath gas analysis for non-invasive detection of various diseases^6–12^. Previous work has shown changes in VOC profiles of cells, which were infected with respiratory syncytial virus (RSV)^10^ and Influenza^6^. Exhaled breath profiles changed after intranasal Influenza A vaccination^13^ in humans. Since the disease takes place in the respiratory tract, VOC profiles exhaled from the lung can be expected to change significantly during Influenza infection. Analysis of breath VOC patterns could, therefore, provide additional and early information on the infection process. A fast and non-invasive diagnosis of infections like Influenza A could be used in human medicine or in animal health control for monitoring in swine or chicken farms. Furthermore, recognizing infections at boarder control may minimize the risk of mixing and rearranging subtypes from different countries.

Changes in VOC profiles may result from pathogens itselves, from interactions between host and pathogen or from host immune responses. To address this issue systematically a complete organism has to be investigated in animal model. Currently, there is not any *in vivo* study on breath gas analysis during a complete infection cycle of Influenza A. Swine show high genetical and physiological similarity to humans, and Influenza infection occurs similarly to humans. Hence, swine offer many advantages compared to other large animal infection models. In earlier experiments, mechanically ventilated swine were used for VOC analyses, as mechanical ventilation enabled reliable breath sampling and constant respiratory rates^14^. In order to be as close as possible to real life screening conditions spontaneously breathing swine without anaesthesia would be a promising biological infection model for VOC analysis during Influenza A infection.

In this pilot study, breath volatiles from spontaneously breathing swine were analyzed during an Influenza A infection by means of NTD pre-concentration and GC-MS analysis.

The following questions were assessed in detail:

- Is breath sampling possible in spontaneously breathing awake swine?
- Are there changes in VOC profiles when animals are tested positive for Influenza A?
- Can VOCs mirror Influenza A infection?

## Results

### Determination of intranasal virus load

Animals reacted differently to intranasal application of the virus suspension on day 0. With respect to virus presence in the nasal cavity from day 2 onwards, swine were defined as not-infected or as Influenza A positive. Animals showed different viral shedding on day 2 and 10 animals were tested positive for Influenza A on day 4 as shown in Tables 1 and 2. From day 7 onwards (day 7 and 14), all animals were tested negative for Influenza A and, thus, had recovered from infection. During the whole experiment, we did not observe any clinical symptoms. The animal in which Influenza A virus could not be detected in nasal swabs on day 4 was excluded from evaluation and statistical analysis on this day. In the control group, tests for Influenza A virus presence were negative during the whole experiment.

**Table 1:** Results of nasal virus test of animals in the infected group during the study period.

Day	0	2	4	7
Swabs tested negative for Influenza A	11	8	1	11
Swabs tested positive for Influenza A	0	3	10	0

**Table 2:** Intranasal virus load on day 2 and 4 from animals of the infected group listed by their ear tag numbers.

Swine	7407	7408	7995	7991	7433	7403	7970	7478	7939	7434
Day 2	6,8 × 10^2^	0	0	0	0	1,4 × 10^3^	0	1,4 × 10^4^	0	0
Day 4	1,5 × 10^4^	1,5 × 10^3^	1,5 × 10^3^	3,2 × 10^3^	1,5 × 10^3^	7,1 × 10^2^	6,8 × 10^3^	1,5 × 10^5^	6,7 × 10	1,5 × 10^4^

### Hematologic analyses

Counts of white blood cells, neutrophils, lymphocytes, monocytes, eosinophils, and basophils did not show any differences between control group and infected group, nor were there mayor changes within the infected group over time as seen in supplementary Table S1.

### VOC analysis

Different criteria were used to select the substances of interest which were then analyzed in more detail. In a first step, compounds with well-known exogenous origin, such as ethanol or 2-butanone being ingredients of disinfectants were excluded from further analysis. In a second step, substances with differences below 10% between control group and infected group and concentrations below LOQ were excluded from statistical analysis and from calibration. In this way, 26 compounds of interest were identified in swines’ breath. Emissions of sampling devices including virus filter and glass syringes had been tested before and had not any influence onto emissions in animals’ breath.

**Figure 1:**
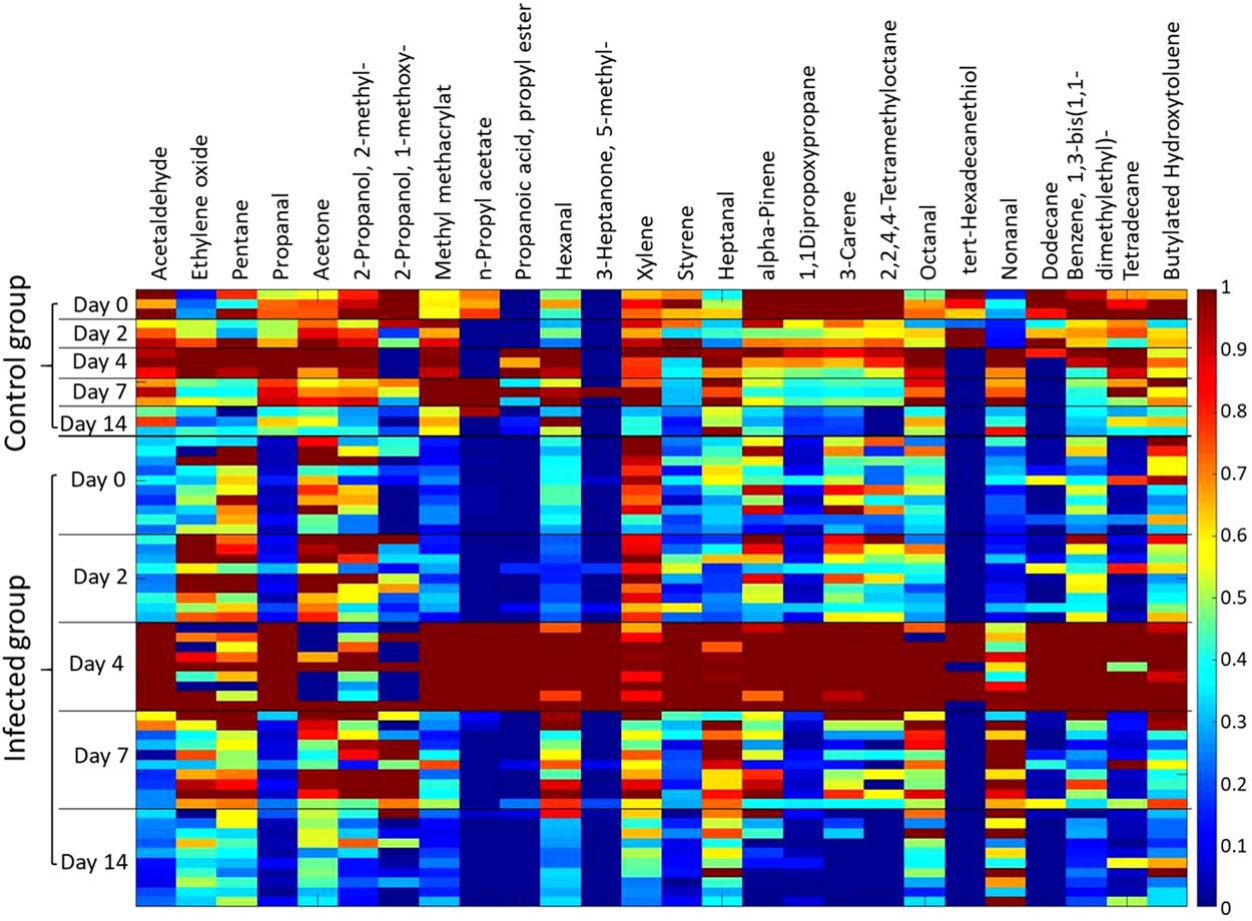
Heatmap: Compound target response of each swine normalized to the individual maximum on day 0, 2, 4, 7, and 14 of measurement.

Changes of substance concentrations from each individual animal are shown in Fig. 1. Most substances in the breath of the infected group reached their maximum on day 4, e.g. acetaldehyde, propanal, and n-propyl acetate.

Statstically significant differences between control and infected group were found for the 6 compounds acetaldehyde, propanal, n-propyl acetate, methyl-methacrylate, styrene, 1,1-dipropoxypropane. Acetone was chosen as additional substance since it is an important marker linked to glucose metabolism and lipolysis.

### Identification and quantification of VOCs in animals‘ breath

For the seven compounds of interest, limits of detection (LOD) were determined from 0.0005 nmol/l to 0.49 nmol/l and limit of quantification (LOQ) from 0.0007 nmol/l to 0.83 nmol/l. Linear ranges were from 0 nmol/l to 10.4 nmol/l as shown in Table 3. Peak areas below LOD were rejected and compound detection between LOD and LOQ are specially indicated in Table 3. Compound concentrations of both groups on each day are shown in supplementary Table S2 as medians with 25^th^ and 75^th^ percentiles. Concentration of room air samples are shown in supplementary Table S3.

**Table 3:** Limit of detection (LOD), limit of quantification (LOQ), standard deviation of the 10 blank needle-traps for determination of LOD and LOQ, and linear range of the seven compounds of interest.

Compound		LOD [nmol/l]	LOQ [nmol/l]	Linear range [nmol/l]
Acetaldehyde	concentration [nmol/l]	0.49	0.83	0–4.2
	Standard deviation	15%		
Propanal	concentration [nmol/l]	0.08	0.12	0–2.1
	Standard deviation	9%		
N-Propyl acetate	concentration [nmol/l]	0.004	0.005	0–10.4
	Standard deviation	7%		
Methyl methacrylate	concentration [nmol/l]	0.04	0.05	0–10.4
	Standard deviation	6%		
Styrene	concentration [nmol/l]	0.05	0.08	0–4.2
	Standard deviation	9%		
1,1-Dipropoxy-propane	concentration [nmol/l]	0.0005	0.0007	—
	Standard deviation	5%		
Aceton	concentration [nmol/l]	0.18	0.23	0–2.1
	Standard deviation	4%		

### Statistical analysis

#### Principal component analysis (PCA)

**Figure 2:**
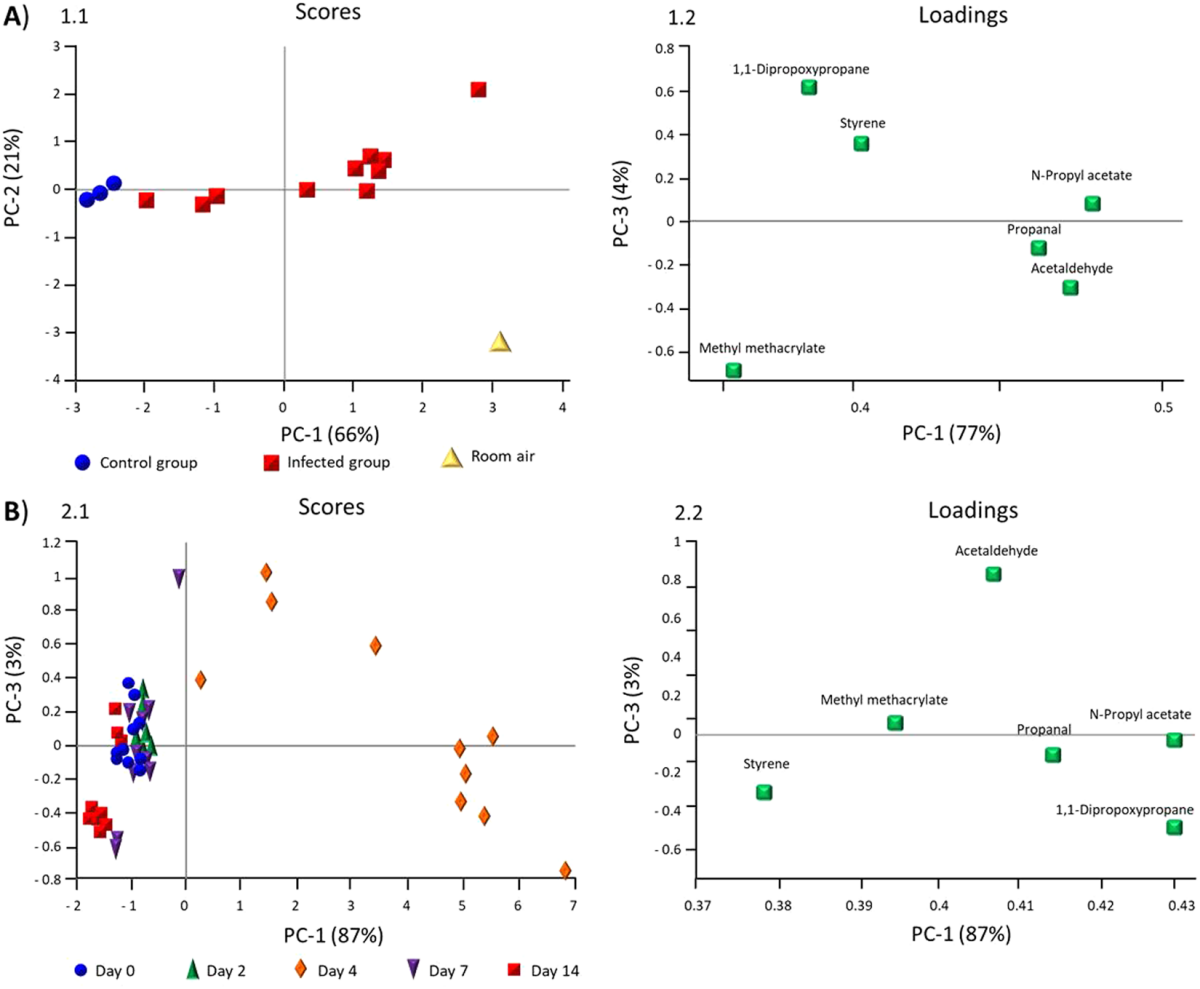
Principal Component Analysis: Scores (1.1, 2.1) and loadings (1.2, 2.2) of VOC emissions. (**A**) Control group (blue) compared to infected group (red), and room air (yellow) on day 4. (**B**) VOC emissions of the infected group on each day of breath measurement.

Results from principal component analysis (PCA) are shown in Fig. 2. The PCA (Fig. 2A1.1) shows that the control group can be separated from the infected group and from room air on day 4 based on changing concentrations of six VOCs. Scores from the control group are located near the y axis, mostly due to concentrations of methyl methacrylate and 1,1 dipropoxypropane. Scores from the infected group are situated in the centre due to the concentrations of n-propyl acetate, propanal, and acetaldehyde (Fig. 2A1.2).

In Fig. 2B2.1 breath samples on day 0 2, 7, and 14 are concentrated near the y axis mostly due to concentrations of styrene and methyl methacrylate. Scores on day 4 can be separated from all other scores mostly due to concentrations of acetaldehyde, n-propyl acetate, 1,1-dipropoxypropane, and propanol (Fig. 2B2.2).

Hence, day 4 can be separated by PCA (Fig. 2B2.1) from all other days of measurement in the infected group. Day 4 was the only day when nasal swabs from all animals were tested positive for Influenza A.

#### Differences between control group and infected group on each day and significant differences between each day of measurement within the infected group

**Figure 3:**
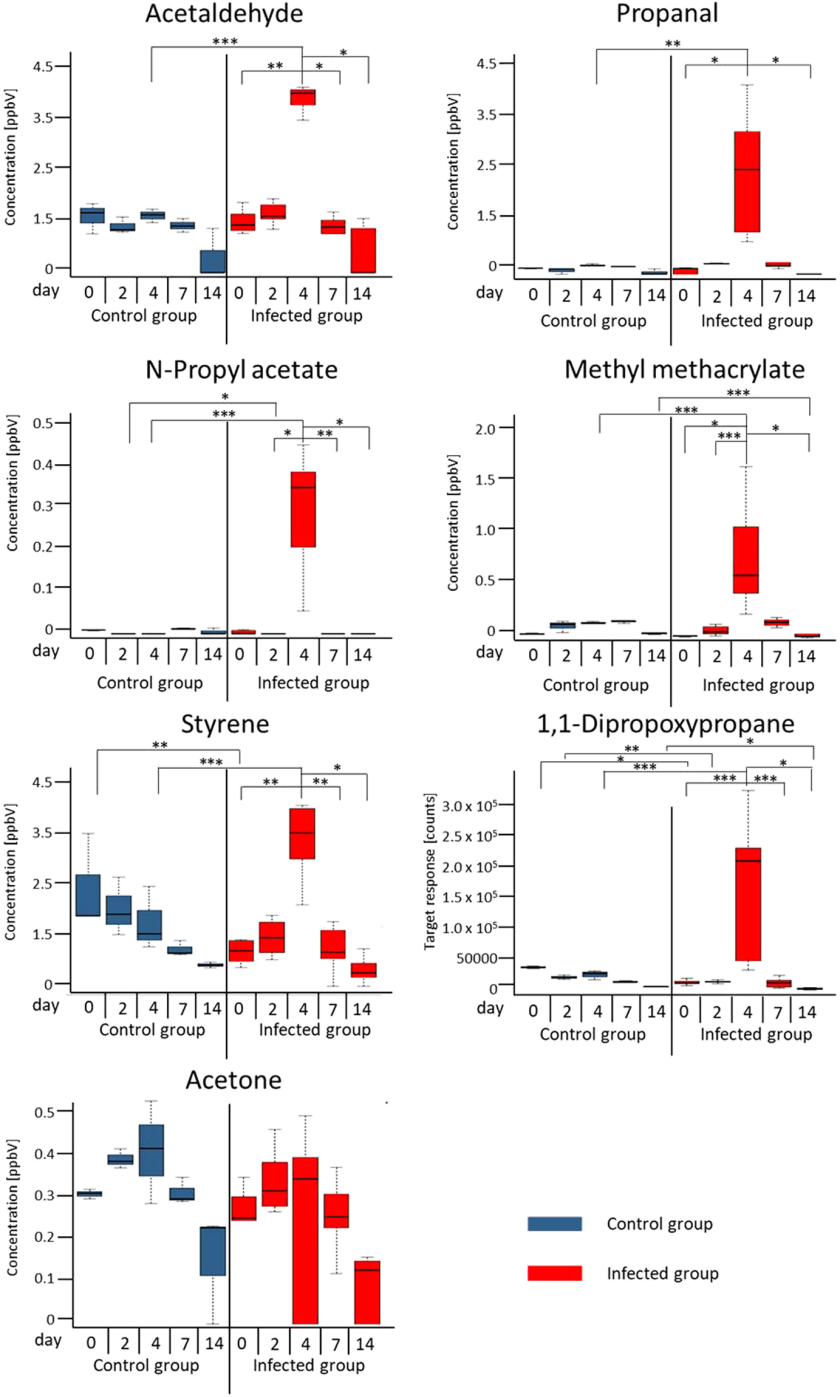
Exhaled concentration of compounds on day 0, 2, 4, 7, and 14 in control (blue) and infected group (red). Significant differences between control group and infected group on each day and significant differences in the infected group between each day *p < 0.001, **p = 0.001-0.01, ***p > 0.01.

Summary of all results are shown as boxplots of quantified concentrations in Fig. 3. Significant differences between the days of VOC analysis within the infected group can be seen in Fig. 3 and Table S4. For all these compounds, day 4 differed clearly from at least three other days of measurement as seen in the boxplot for propanal. The seven compounds of interest showed a significant increase on day 4 when animals were tested positive for Influenza A. Concentrations of these VOCs decreased again after day 4 and were comparable to concentrations in the control group on day 7 and 14. Significant differences between infected group and control group are shown in Table S5.

#### Correlation analysis virus load and VOC concentration

Linear correlations between acetaldehyde and methyl methacrylate concentrations and intranasal virus loads were found. Correlation coefficients were R = 0.71, and R = 0.76 respectively. The other compounds of interest did not show any correlation between VOC concentrations and intranasal virus loads.

## Discussion

Reproducible breath sampling from spontaneously breathing swine was realized despite complex infection control protocols under high safety conditions. Breath VOCs in the low parts per billion concentration range could successfully be determined by means of optimized micro-extraction based pre-concentration, gas chromatography and mass spectrometry during a whole Influenza A infection period of 14 days. VOC profiles were significantly different between control and infected animals. As early as on day 4 post inoculation, infected animals could be separated from not infected controls on the basis of concentration changes of six selected VOCs.

Within the infected group, substance concentrations on day 4 differed significantly from those determined on all other days of the study period. Acetaldehyde und methyl methacrylate showed linear correlations with intranasal virus loads.

Unequivocal identification and reliable substance quantification is mandatory in order to decide which volatile emissions may be linked to infection. Hence, identification of potential marker substances was confirmed by means of analysis of pure reference substances and did not solely rely on database (e.g. NIST) search. Any quantification was done through calibration with reference materials. All breath samples were taken under CO_2_ control in order to unequivocally identify alveolar phases of expiration as substance concentrations in breath only reflect concentrations in blood if alveolar breath is analysed. In addition, alveolar breath sampling significantly reduces variability induced through dilution by dead space air^15^.

Due to the low age of the animals (seven weeks) and their inevitable excitement during breath sampling, they had high respiratory rates and low tidal volumes. Hence, variance of data can be attributed to those effects. In order to reduce the stress level animals were put into a canvas sling during sampling to suppress the behavioural reflex of screaming and flight, which is triggered when piglets are manually held down and freedom of movement is limited.

Different intranasal virus loads in the animals can be explained through individually different immune response after intranasal virus application. While other studies describe lymphopenia^16^ or neutrophilia^17^ in humans as well as lymphopenia and granulocytosis in ferrets during Influenza A infection^18^, we did not see any changes in white blood cell counts of the swine in this study. Since swine in this study did not show any clinical symptoms neither, it might be tempting to speculate that their immune response was by nature lower than reactions in humans or ferrets. Nevertheless, profiles of exhaled VOCs showed characteristic changes during the virus infection cycle. These changes apparently did not mainly depend on an inflammatory host reaction.

Increased exhaled acetaldehyde concentrations on day 4 may be due to inflammatory processes occurring in the airways during Influenza A infection. Acetaldehyde has been linked to inflammatory processes in the respiratory tract and can be generated by leucocytes. High acetaldehyde concentrations were detected in breath of patients suffering from asthma, cancer, or acute respiratory distress syndrome^19^.

Interaction of Influenza A with the natural bacterial population in the upper respiratory tract^2^ could represent a second source of acetaldehyde. Staphylococcus aureus is one of the most common bacterial species in the human respiratory tract and was implicated with emissions of acetaldehyde in the past^20–22^. As similarities between swine and humans also exist concerning the microbiome of the respiratory tract^23^, acetaldehyde production could increase due to interactions between *S. aureus* and the Influenza virus.

Propanal increased significantly in swines’ breath on the day 4 indicating infection induced oxidative stress which is known to trigger lipid peroxidation^13^. Propanal is one of the potential end products of the ω − 3 lipid peroxidation. Increased concentrations of n-propyl acetate have been found in human fibroblasts (HFB), hepatocellular carcinoma cells (HepG2), and primary human bronchial epithelial cells (HBEpC)^24–26^ potentially indicating altered activity and metabolism after virus binding to epithelial cells.

N-propyl acetate may originate from acid catalysed reaction of acetic acid with 2-propanol which is an ubiquitious ingredient of disinfectants. On the other hand, this compound may also be linked to the Influenza A infection. Increased concentration of n-propyl acetate were detected in human fibroblasts (HFB), hepatocellular carcinoma cells (HepG2), and primary human bronchial epithelial cells (HBEpC) cells while the origin of this compound is yet unclear^24,25^. Increased n-Propyl acetate concentration from HBEpC was detected in normal bronchial epithelial cells. Thus, increasing emissions of n-Propyl acetate may be attributed to altered cell activity and metabolism induced through binding of Influenza A to epithelial sialic acid receptors in the respiratory tract.

1,1-Dipropoxypropane is the acetale of 1-propanol and propanal^27^. Since 1-propanol is a common component of disinfectants and propanal was detected in the breath samples it might be possible that 1,1-dipropoxypropane is the product derived from those two compounds during storage of breath samples in the glass syringe.

Methyl methacrylate production is linked to bacteria such as *Staphylococcus aureus* and *Pseudomonas aeruginosa*^20,21^. In humans, it is known that these bacteria often belong to the natural mucosal population of the upper respiratory tract. Based on the genetic and physiological similarities between humans and swine this natural bacterial population is also present in healthy swine^23^. Since it is already known that Influenza infection also affects the bacterial population in the respiratory tract^2^, increased emission of methyl methacrylate could occur from increased interactions between Influenza A virus and *S. aureus* and *P. aeruginosa*.

In earlier studies, styrene has been detected in breath and has been linked to diseases such as cancer^28^ and asthma^29^. In a recent investigation styrene and 1,3-bis(1,1-dimethylethyl)-benzene were detected in cells infected with respiratory syncytial virus (RSV)^10^. Both substances were also detected in this study. As styrene has been described as a product from industrial processes^29^ being ubiquitously present in the body^30^ its role as a specific marker seems doubtful.

A traceable correlation between VOC concentrations and intranasal virus load was only found for acetaldehyde and methyl methacrylate. The fact, that this correlation was also valid for the period after day 4 has to be attributed to VOC generation induced by still ongoing immune processes in the body even though the virus could not be detected in the nasal swabs anymore.

With the exception of propanal, VOC emissions showing correlations with Influenza A infection seem to be linked to virus-cell and virus-microbiome interactions rather than to inflammatory host reactions. Given the minimal immune response of the animals it is not surprising that the only trace of a host reaction was an increased exhalation of propanal. These findings suggest, that detection of virus presence might be possible even if there is no or only minimal host reaction. Regarding the fact that animals in this study did not even show fatigue or reduced food intake and had unchanged counts of immune cells in peripheral blood, VOC analysis might create awareness of an otherwise overlooked presence of Influenza virus.

The optimized set-up for sampling infectious breath used in this study enabled VOC analysis from spontaneously breathing swine during an entire Influenza A infection cycle for the first time. For this purpose well-adapted methods such as NTME and a well-controlled *in vivo* setting were used^31–33^. VOC based complimentary information could enlarge our knowledge on virus infection mechanisms in terms of elucidating virus-cell or virus-microbiome interactions.

As Influenza A virus caused characteristic changes in VOC profiles even in the absence of clinical signs or measurable immune pathology, non-invasive breath analysis could enable detection of the virus before any infectious symptoms may be visible in animals or humans. Analysis of VOCs in breath is completely non-invasive and could also be used for large-scale screening purposes.

## Material and Methods

### Ethical statement

The ethics committee of the State Office for Agriculture, Food Safety and Fishery in Mecklenburg-Western Pomerania (LALFF M-V) approved animal experiments in swine with the reference number 7221.3-1-035/17. All procedures were carried out in accordance with the relevant guidelines and regulations.

### Cells and virus

Influenza A Virus A/Bayern/74/2009 (H1N1pdm09) (By09) was propagated on Madin-Darby canine kidney cells (MDCK II) cells in MEM containing 0.2% bovine serum albumin, 1 Unit/ml Penicillin, 1 µg/ml Streptomycin and 2 µg/ml N-tosyl-L-phenylalanine chloromethyl ketone (TPCK)-treated trypsin (Sigma-Aldrich). For TCID_50_ assay, serial tenfold dilutions in infection medium were prepared and added to MDCK II cells on 96-well tissue culture plates. After incubation for three days at 37 °C and 5% CO_2_ each well was monitored for cytopathic effects. Viral titers were calculated according to Spearman-Kärber^34^.

### Infection of swine and sample protocol

**Figure 4:**
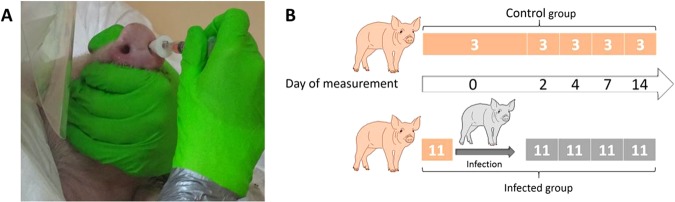
(**A**) Intranasal inoculation process in swine. Animals were inoculated intranasally at seven weeks of age by MAD with 2 ml By09. (**B**) Sample protocol of breath gas analysis and determination of intranasal virus load. Breath samples and nasal swabs for virus determination were taken from each animal on day 0, prior to infection. After intranasal inoculation of 11 animals (infected group) with Influenza A on day 0, breath samples and virus load of the infected animals and the three control animals was determined by virus titration on MDCK II cells.

Fourteen seven-week old German landrace swine were obtained from a commercial high health status herd and were separated randomly in three different sheds at the federal research institute for animal health, Friedrich-Loeffler-Institut on the island of Riems (Germany). One shed was used only for the three control animals and the other two for the infection group. All animals were tested negative for acute Influenza virus infection by matrix gene quantitative RealTime-PCR (AgPath.ID™ One-Step RT-PCR Kit, Applied Biosystems) on nasal swabs (DRYSWAB™, mwe, UK) prior to infection (modified from^35^). On the first day (day 0), prior to inoculation breath samples were taken from all animals. Then, eleven swine were infected intranasally at seven weeks of age by mucosal atomization device (MAD) (Prosys International Ltd) with 2 ml By09 (infected group) as seen in Fig. 4A. Additional breath samples and nasal swabs were taken from every group on day 2, 4, 7, 14, 21 after inoculation as shown in Fig. 4B.

### Determination of intranasal virus load

VOC concentrations in breath and virus load in animals’ nasal cavity were always determined simultaneously. Swabs taken from each nostril were placed in media directly after sampling and incubated at room temperature on a shaker for 2 hours to elute the virus. After squeezing and discarding the swab, TCID_50_ assays of supernatants were carried out as described above.

### Hematological analyses

Count of different immune cells in the blood was determined from freshly collected EDTA-blood samples from swine in an automated hematology analyzer (Abbott Cell-Dyn 3700) on each day of sampling. Absolute numbers of white blood cells (WBC), lymphocytes, monocytes and neutrophil, eosinophil and basophil granulocytes were determined.

### VOC analysis

#### Breath sampling

**Figure 5:**
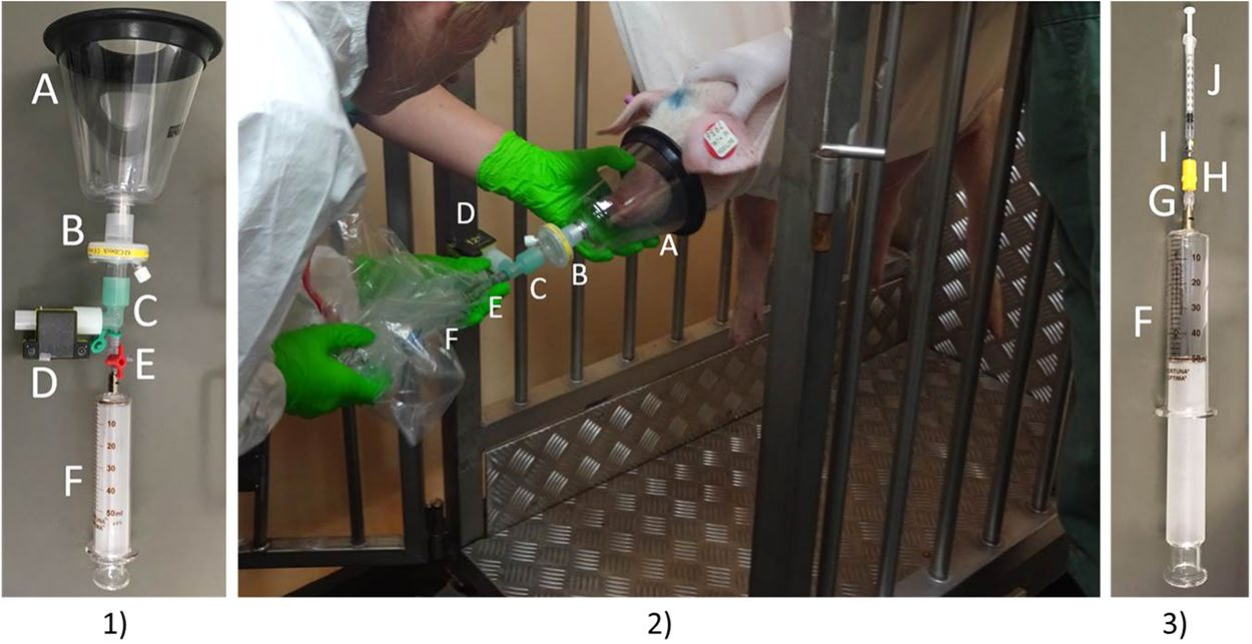
Breath sampling under high safety conditions. (1) breath sampling device, (2) breath sampling from swine in canvas sling, and (3) bi-directional NTD-sampling. A = breathing mask, B = virus-bacteria filter, C = T-piece, D = capnometer, E = three-way cock, F = 50 ml glass syringe, G = female/female adapter, H = IN stopper, I = Needle-trap, J = 1 ml syringe.

After placing the animals into a canvas sling^36^, a dog breathing mask (Henry Schein Medical Austria GmbH, Wien, Austria) was donned to the snout. This mask was connected to a virus-bacteria filter (Wolfram Droh GmbH, Mainz, Germany), which was connected to a three-way cock (Discofix® B. Braun Melsungen AG, Germany) and a T-piece. A 50 ml glass syringe (F in Fig. 5-1), FORTUNA® OPTIMA®, Germany) was linked to the T-piece and after flushing the set-up with one breath cycle, 50 ml of animals’ breath were taken under capnometer (EMMA Masimo, Irvna, USA) control. After sampling, the three-way cock was closed and exchanged for a female/female adapter (B Braun, Melsungen, Germany) which was connected to an IN stopper (B Braun, Melsungen, Germany). The glass syringe was kept in a plastic bag (RUBIN EasyZip 3 l, Dirk Rossmann GmbH, Burgwedel) for the whole sampling process to avoid broken glass in the pigsty in case of damages or dropping the syringe as shown in Fig. 5-2). Additionally, the emissions of the set-up system including virus-bacteria filter were tested. For this purpose 50 ml Helium were sampled with a 50 ml glass syringe and analyzed by means of NTME-GC-MS analysis. After breath sampling from the animals a room air sample was drawn through the sampling device (mask and filter). The set-up is shown in Fig. 5.

#### VOC NTD-Preconcentration

For VOC preconcentration copolymer methacrylic acid and ethylene glycol dimethacrylate needle trap devices (NTDs) were used. Preparation and preconditioning of NTDs were done as described before^37^. NTDs were connected to 1 ml syringe (J) (Tuberkulin, B Braun) and jabbed trough the IN-Stopper (H) to the inside of the 50 ml glass syringe (F) as shown in Fig. 5-3). VOCs from headspace in the syringe were preconcentrated by sampling 20 ml bi-directionally as described before^37^.

#### VOC analysis by GC-MS

For VOC analysis, an Agilent 7890 A gas chromatograph coupled to an Agilent 5975 C inert XL MSD with triple axis detector was used. Compounds on NTDs were thermally desorbed at an injector temperature of 200 °C in splitless mode for 60 s. Separation was done with a 60 m RTX- 624 column (0.32 mm ID, 1.8 μm column thickness) using a constant carrier gas (He) flow of 1.5 ml/min and the following temperature program: 40 °C for 5 min, 8 °C/min to 120 °C for 2 min, 10 °C/min to 220 °C and 20 °C/min to 240 °C for 4.5 min. Mass spectrometric VOC detection was done by means of electron impact ionization (EI −70 eV) in full scan mode with mass range 35–250 amu and scan rate 2.73 scan/s as previously described^37^.

#### Calibration

After tentative identification of mass spectral peaks by means of NIST (Version 2.0) spectral library confirmation of substance identity and calibration were achieved by means of pure reference substances for acetaldehyde, propanal, n-propyl acetate, methyl methacrylate, styrene and acetone. 1,1-Dipropoxypropane was only identified through NIST database.

For quantification of acetaldehyde, styrene, and acetone a VOC gas standard mixture (Ionicon Analytik GmbH, Innsbruck, Austria) was used. A calibration curve with 5 calibration steps from 0.5 ppbV to 100 ppbV was prepared using a liquid calibration unit LCU (Ionicon Analytik GmbH, Innsbruck, Austria). A liquid calibration solution was prepared with the reference standards propanal, n-propyl acetate, and methyl-methacrylat (Sigma Aldrich, Darmstadt, Germany) with 5 calibration steps from 0.02 nmol/l (0.5 ppbV) to 10.4 nmol/l (250 ppbV). 1,1-Dipropoxypropane was not available as reference substance and could, therefore, not be calibrated.

Three 50 ml glass syringes of each calibration level were heated up to 50 °C, filled with 50 ml standard gas directly from the LCU and closed with an IN-stopper. Pre-concentration of volatile substances was achieved through bidirectional sampling of 20 ml from the syringes in the same way as during breath sampling. LOD and LOQ were determined by analyzing 10 blank samples as described before^37^.

### Statistical analysis

For creating heatmaps, MATLAB Software (R2017a) was used. For principal component analysis (PCA) “The Unscrambler X” software (version 10.3) was used. Statistical testing of significant differences between the control group and the infected group and between different days was done by SigmaPlot software (version 13.0) using the Normality Test (Shapiro-Wilk), the Equal Variance Test (Brown-Forsythe), and the Kruskal-Wallis One Way Analysis of Variance on Ranks. Boxplots and correlations analysis (PEARSON) were done using R software (version 3.3.2_ 2016-10-31) and RStudio (version 1.0.136).

## Electronic supplementary material

Supplementary Information

## Data Availability

We comply with data availability policy of this journal.
