# Supplementary material for: VOC breath profile in spontaneously breathing awake swine during Influenza A infection
Source: Sci Rep. 2018 Oct 5;8:14857. doi: 10.1038/s41598-018-33061-2 (PMC6173698; doi:10.1038/s41598-018-33061-2)
Supplement: Supplementary file 1 — Supplementary Information [file 41598_2018_33061_MOESM1_ESM.docx]

# VOC breath profile in spontaneously breathing awake swine during Influenza A infection

Selina Traxler^1^, Ann-Christin Bischoff^1^, Radost Saß^1^, Phillip Trefz^1^, Peter Gierschner^1^, Beate Brock^1^, Theresa Schwaiger^2^, Claudia Karte^3^, Ulrike Blohm^4^, Charlotte Schröder^2^ Wolfram Miekisch^1^* and Jochen K Schubert^1^

^1^ Department of Anaesthesiology and Intensive Care, Rostock University Medical Center, ROMBAT, Schillingallee 35 18057 Rostock, Germany

^2^ Department of Experimental Animal Facilities and Biorisk Management, Friedrich-Loeffler-Institute, Südufer 10, 17493 Greifswald- Insel Riems

^3^ Institute of Diagnostic Virology, Friedrich-Loeffler-Institute, Südufer 10, 17493 Greifswald-Insel Riems

^4^ Institute of Immunology, Friedrich-Loeffler-Institute, Südufer 10, 17493 Greifswald-Insel Riems

* [wolfram.miekisch@uni-rostock.de](mailto:wolfram.miekisch@uni-rostock.de)

## Supplementary Information

Table S 1: Determined of number of cells x 1000 per µl whole blood of the control group on day 0 and the infected group on each day of measurement

| group | day | white blood cells | Neutrophils | Lympho-cytes | Monocytes | Eosinophils | Basophils |
| --- | --- | --- | --- | --- | --- | --- | --- |
| Control group | 0 | 19.70 | 9.83 | 9.10 | 0.537 | 0.270 | 0.005 |
|  | 0 | 9.33 | 5.09 | 3.67 | 0.256 | 0.280 | 0.037 |
|  | 0 | 17.80 | 6.99 | 10.60 | 0.121 | 0.119 | 0.002 |
| Infected group | 0 | 20.5 | 10.5 | 9.08 | 0.354 | 0.579 | 0.026 |
|  | 0 | 20.0 | 9.88 | 9.19 | 0.327 | 0.608 | 0.032 |
|  | 0 | 19.0 | 10.6 | 7.42 | 0.584 | 0.338 | 0.018 |
|  | 0 | - | - | - | 0.026 | 0.051 | 0.00 |
|  | 0 | 11.9 | 4.86 | 6.76 | 0.101 | 0.140 | 0.010 |
|  | 0 | 14.9 | 6.98 | 7.54 | 0.066 | 0.297 | 0.00 |
| Infected group | 2 | 14.7 | 6.72 | 7.54 | 0.249 | 0.171 | 0.032 |
|  | 2 | 12.5 | 4.71 | 7.03 | 0.293 | 0.350 | 0.072 |
|  | 2 |  | - | 3.89 | 0.193 | 0.240 | 0.019 |
|  | 2 | 11.3 | 4.92 | 5.09 | 0.429 | 0.828 | 0.003 |
|  | 2 | 12.9 | 5.63 | 6.92 | 0.216 | 0.107 | 0.009 |
|  | 2 | 12.0 | 4.57 | 6.89 | 0.346 | 0.189 | 0.030 |
| Infected group | 4 | 15.4 | 7.52 | 7.05 | 0.642 | 0.129 | 0.092 |
|  | 4 | 12.4 | 6.61 | 4.99 | 0.555 | 0.229 | 0.029 |
|  | 4 | 19.9 | 12.6 | 5.50 | 1.17 | 0.571 | 0.032 |
|  | 4 | 18.0 | 11.0 | 4.85 | 1.54 | 0.408 | 0.188 |
|  | 4 | 13.8 | 5.14 | 8.19 | 0.336 | 0.159 | 0.008 |
|  | 4 | 13.9 | 7.49 | 6.15 | 0.065 | 0.245 | 0.00 |
| Infected group | 7 | 12.6 | 6.74 | 5.37 | 0.115 | 0.417 | 0.008 |
|  | 7 | 13.8 | 6.70 | 6.66 | 0.116 | 0.342 | 0.00 |
|  | 7 | 14.8 | 8.61 | 5.88 | 0.132 | 0.183 | 0.00 |
|  | 7 | 16.9 | 8.26 | 8.09 | 0.201 | 0.343 | 0.00 |
|  | 7 | 11.0 | - | 6.24 | 0.684 | 0.104 | 0.190 |
|  | 7 | 14.5 | 7.70 | 6.34 | 0.145 | 0.319 | 0.002 |
| Infected group | 14 | 11.2 | 3.84 | 6.63 | 0.353 | 0.301 | 0.094 |
|  | 14 | 14.1 | 5.31 | 7.64 | 0.701 | 0.278 | 0.196 |
|  | 14 | 14.8 | 6.81 | 7.14 | 0.524 | 0.297 | 0.035 |
|  | 14 | 17.6 | 9.56 | 6.36 | 1.01 | 0.430 | 0.210 |
|  | 14 | 14.0 | 6.72 | 6.77 | 0.268 | 0.196 | 0.019 |
|  | 14 | 14.5 | 6.65 | 6.96 | 0.416 | 0.368 | 0.113 |

Table S 2: Median, 25^th^ percentile, 75^th^ percentile, and detected compounds below LOQ from compounds on day 0, 2, 4, 7, and 14 in breath of animals from the control group and the infected group

| Compound | Day | **Control group** | | | | | **Infected group** | | | | |
| --- | --- | --- | --- | --- | --- | --- | --- | --- | --- | --- | --- |
|  |  | 0 | 2 | 4 | 7 | 14 | 0 | 2 | 4 | 7 | 14 |
| Acetaldehyde | Median [nmol/l] | 1.3 | 0.9 | 1.3 | 1.0 | 0.5 | 1.0 | 1.2 | 3.9 | 1.0 | 1.0 |
|  | 25^th^ percentile | 1.1 | 0.9 | 1.2 | 0.9 | 2.4 | 0.9 | 1.2 | 3.6 | 1.0 | 0 |
|  | 75^th^ percentile | 1.4 | 1.1 | 1.3 | 1.1 | 0.7 | 1.3 | 1.5 | 4.0 | 1.1 | 1.0 |
|  | Detected compounds below LOQ |  |  |  |  | 1 |  |  |  | 1 | 5 |
| Propanal | Median [nmol/l]] | 0.1 | 0.1 | 0.2 | 0.2 | 0.1 | 0.1 | 0.3 | 2.3 | 0.2 | 0 |
|  | 25^th^ percentile | 0.1 | 0.1 | 0.2 | 0.2 | 0 | 0.1 | 0.2 | 0.9 | 0.2 | 0 |
|  | 75^th^ percentile | 0.1 | 0.1 | 0.2 | 0.2 | 0 | 0.1 | 0.3 | 2.5 | 0.3 | 0 |
|  | Detected compounds below LOQ |  | 1 |  |  | 2 | 2 |  |  |  | 6 |
| N-Propyl acetate | Median [nmol/l] | 0.008 | 0 | 0 | 0.01 | 0 | 0.003 | 0 | 0.3 | 0 | 0 |
|  | 25^th^ percentile | 0.007 | 0 | 0 | 0.01 | 0 | 0 | 0 | 0.2 | 0 | 0 |
|  | 75^th^ percentile | 0.008 | 0 | 0 | 0.01 | 0.006 | 0.007 | 0 | 0.3 | 0 | 0 |
|  | Detected compounds below LOQ |  |  |  |  |  | 1 |  |  |  |  |
| Methyl methacrylate | Median [nmol/l] | 0.2 | 0.3 | 0.3 | 0.3 | 0.2 | 0.1 | 0.2 | 0.7 | 0.3 | 0.1 |
|  | 25th percentile | 0.2 | 0.2 | 0.3 | 0.3 | 0.2 | 0.1 | 0.1 | 0.5 | 0.2 | 0.1 |
|  | 75th percentile | 0.2 | 0.3 | 0.3 | 0.3 | 0.2 | 0.1 | 0.2 | 1.1 | 0.3 | 0.2 |
| Styrene | Median [nmol/l] | 1.6 | 1.7 | 1.2 | 0.8 | 0.5 | 0.8 | 1.1 | 3.4 | 0.8 | 0.3 |
|  | 25th percentile | 1.6 | 1.4 | 1.1 | 0.8 | 0.5 | 0.6 | 0.8 | 3.0 | 0.6 | 0.2 |
|  | 75th percentile | 2.6 | 2.1 | 1.7 | 0.9 | 0.5 | 1.0 | 1.5 | 4.1 | 1.2 | 0.5 |
|  | Detected compounds below LOQ |  |  |  |  | 1 | 4 |  |  | 3 | 6 |

Table S 2: Median, 25^th^ percentile, 75^th^ percentile, and detected compounds below LOQ from compounds on day 0, 2, 4, 7, and 14 in breath of animals from the control group and the infected group

| Compound | Day | **Control group** | | | | | **Infected group** | | | | |
| --- | --- | --- | --- | --- | --- | --- | --- | --- | --- | --- | --- |
|  |  | 0 | 2 | 4 | 7 | 14 | 0 | 2 | 4 | 7 | 14 |
| Aceton | Median [nmol/l] | 0.3 | 0.4 | 0.4 | 0.3 | 0.2 | 0.3 | 0.3 | 0.4 | 0.3 | 0 |
|  | 25^th^ percentile | 0.3 | 0.4 | 0.4 | 0.3 | 0.1 | 0.3 | 0.3 | 0 | 0.2 | 0 |
|  | 75^th^ percentile | 0.3 | 0.4 | 0.5 | 0.3 | 0.2 | 0.3 | 0.4 | 0.4 | 0.3 | 0 |
|  | Detected compounds below LOQ |  |  |  |  |  | 2 |  | 1 | 1 | 4 |

Table S 3: Compound concentration [nmol/l] in room air

| Day | 0 | 2 | 4 | 7 | 14 |
| --- | --- | --- | --- | --- | --- |
| Acetaldehyde | 0.7 | 0.8 | 7.6 | 0.8 | 0 |
| Propanal | 0.2 | 0.3 | 4.2 | 0.3 | 0 |
| n-Propyl acetate | 0 | 0 | 0.3 | 0 | 0 |
| Styrene | 1.4 | 1.7 | 4.9 | 0.9 | 0.4 |
| Methyl methacrylat | 0.2 | 0.2 | 5.0 | 0.3 | 0.2 |
| Aceton | 0.5 | 0.4 | 0 | 0.3 | 0 |

Table S 4: Differences in the infected group between days of measurement

| Compound | Day 4 vs. 0 | | Day 4 vs. 2 | | Day 4 vs. 7 | | Day 4 vs. 14 | |
| --- | --- | --- | --- | --- | --- | --- | --- | --- |
| Acetaldehyde | s. | P=0.008 | n.s. | - | s. | P<0.001 | s. | P<0.001 |
| Propanal | s. | P<0.001 | n.s. | - | n.s. | - | s. | P<0.001 |
| N-Propyl acetate | n.s. | - | s. | P<0.001 | s. | P=0.002 | s. | P<0.001 |
| Methyl methacrylate | s. | P<0.001 | s. | P=0.022 | n.s. | - | s. | P<0.001 |
| Styrene | s. | P=0.005 | n.s. | - | s. | P=0.004 | s. | P<0.001 |
| 1,1-Dipropoxy-propane | s. | P=0.013 | n.s. | - | s. | P=0.011 | s. | P<0.001 |

s.: significant difference, n.s.: no significant difference

Table S 5: Differences between infected group and control group

| Compound | Day 0 | | Day 2 | | Day 4 | | Day 7 | Day 14 | |
| --- | --- | --- | --- | --- | --- | --- | --- | --- | --- |
| Acetaldehyde | n.s. | - | n.s. | - | s | P=0.011 | n.s. | n.s. | - |
| Propanal | n.s. | - | - |  | s | p=0.002 | n.s. | n.s. | - |
| N-Propyl acetate | n.s. | - | s. | P<0.001 | s | p=0.013 | n.s. | n.s. | - |
| Methyl methacrylate | n.s. | - |  |  | s | P=0.039 |  | s. | p=0.018 |
| Styrene | s. | p=0.005 | n.s. |  | s. | p=0.018 | n.s. | n.s. | - |
| 1,1-Dipropoxy-propane | s. | p<0.001 | s. | p=0.005 | s. | p=0.039 | n.s. | s. | p<0.001 |

s.: significant difference, n.s.: no significant difference
